# Epidemiology and factors associated with diarrhoea amongst children under 5 years of age in Engela district in the Ohangwena region, Namibia

**DOI:** 10.4102/phcfm.v12i1.2361

**Published:** 2020-08-24

**Authors:** Maria F. Bauleth, Honore K. Mitonga, Lusia N. Pinehas

**Affiliations:** 1 School of Nursing, Faculty of Health Sciences, University of Namibia, Oshakati, Namibia; 2 School of Public Health, Faculty of Health Sciences, University of Namibia, Oshakati, Namibia

**Keywords:** COVID-19, pandemic, Nigeria, family physicians, frontline

## Abstract

**Background:**

Diarrhoea remains a public health problem and an important cause of morbidity and mortality amongst children, mainly in low- and middle-income countries. In Namibia, the national prevalence of diarrhoea was 17%; it was responsible for 5% of all deaths in children under 5 years old and is the second leading cause of death.

**Aim:**

The purpose of this study was to assess the epidemiology and factors associated with acute diarrhoea amongst children less than 5 years of age in Engela district in the Ohangwena region, Namibia.

**Setting:**

The study was conducted in Ohangwena Region in Namibia which extends east to west along the borders of the southern part of Angola.

**Methods:**

A cross-sectional study was conducted. A structured questionnaire was administered through face-to-face interviews. Descriptive statistics were used to describe the socio-demographic and epidemiological data of diarrhoea and logistic regression analysis was used to determine the factors associated with the prevalence of diarrhoea.

**Results:**

The study found a prevalence of 23.8% for diarrhoea in the 2 weeks period preceding the survey amongst children aged under 5 years. The prevalence of diarrhoea was statistically significantly associated with children (*p* < 0.05). The strongest predictor of the prevalence of diarrhoea was the residential area ‘informal settlement’, with an odds ratio of 36.42. This implies that children living in the informal settlement are 36.42 times at risk of contracting diarrhoea as compared to those living in other residential areas.

**Conclusion:**

epidemiology; factors; diarrhoea; under-5 years children; Engela district; Ohangwena region; Namibia.

## Background

Diarrheal disease is ranked as the second most common cause of death amongst children under 5 years of age, leading to an estimated 1.87 million deaths globally.^1^ More than half a million children under 5 were estimated to have died from diarrhoeal disease in 2015.^2^ Diarrhoea remains a public health problem in developing countries, accounting for more than 760 000 deaths of children aged under 5 every year, in both low- and middle-income countries.^3,4^ The majority of deaths take place in Africa and South Asia; however, nearly half of those deaths occur in Africa.^1,5^ Even though over the past 25 years mortality from diarrhoea in children aged under 5 years has declined considerably worldwide, in sub-Saharan Africa morbidity from diarrhoeal disease remains high as a result of inadequate water, poor sanitation or hygiene, insufficient breast feeding and malnutrition.^6^ Increased internal migration to African cities results in overcrowding and is often associated with outbreaks of diarrhoea amongst children under the age of 5 years. Children aged under 5 are the most vulnerable to diarrheal disease, especially during the first 2 years of life.^7^ Various studies have indicated that epidemiologic factors that contribute to the occurrence of diarrhoea are complex.^8,9^ Nevertheless, factors such as residential area, unemployment, household income, mother or caregiver age, number of people per household, access to information, type of toilet facilities, access to safe drinking water, child immunisation status, nutritional status and number of sleeping rooms have been reported to contribute to diarrhoeal prevalence.^7,10,11,12^ According to a study conducted by Thiam et al.^6^ in Mbour, Senegal, factors such as unemployment of mothers, use of shared toilets and no treatment of stored drinking water were found to be significantly associated with diarrhoeal prevalence. Equally, according to a cross-sectional study conducted in Eastern Ethiopia, major risk factors for diarrhoea included improper waste disposal practices, lack of handwashing facilities, living in rural areas, andpresence of two or more siblings in a household aged under 5 were found to be significantly associated with the prevalence of diarrhoea.^7^ Based on a study conducted in Bangladesh on the prevalence and healthcare-seeking behaviours for diarrhoeal disease, several factors such as age of the child, age-specific height, age and occupation of the mothers, residential area and the type of toilet facilities were found to be significantly associated with the prevalence of diarrhoea predicted directly by crude odds ratios (ORs).^4^ It has been reported that several countries have successfully used mass media, especially radio and television, to spread regular messages on breastfeeding and advocacy for policy support of community-driven development (CDD) programmes.^4^ The possibility of reducing morbidity and mortality related to diarrhoea requires well-informed parents. Some cases of diarrhoea are caused by vaccine-preventable pathogens; therefore, children’s immunisation plays a vital role in the prevention of diarrhoea.^13^

In Namibia, especially in Ohangwena region, diarrhoeal disease is estimated to affect 19% of children under the age of 5.^14^ According to Ohangwena Health Directorate Annual Report of 2016 and 2017, 22 201 children under 5 years were diagnosed with diarrhoea, of which 11 507 (52%) were from Engela Health District where the current study took place. Similarly, it is estimated that 62.3% of the population in the Ohangwena region lives in the Engela district. This massive number of population in the district resulted in the spread of informal settlements and lack of basic services, such as water provision, solid waste removal and lack of toilet facilities. Such living conditions are reported to create a high risk for water-borne and gastrointestinal diseases including diarrhoeal diseases.^6^

In Namibia, despite the high prevalence of diarrhoea, there are limited reports from population-based studies. Moreover, to better understand the factors associated with prevalence of diarrhoea in these settings, data are needed. Such data will be valuable for planning, designing, implementation of interventions and prevention strategies targeted at decreasing morbidity because of diarrhoea at the community level. Thus, the objective of this study was to assess the epidemiology of diarrhoea and associated factors amongst children under 5 years old.

## Methods

### Study design and study site

A community-based cross-sectional study was conducted in Engela district in Ohangwena region between January 2019 and March 2019. The study site is approximately 738 km from Windhoek, the capital of Namibia. Ohangwena region is situated in the northern part of the country and connects its borders with the Cunene province as well as part of Cuando Cubango province in the southern part of Angola.^15^ Out of the 11 constituencies in the Ohangwena region, seven (Ongenga, Endola, Ondobe, Oshikango, Omulondo, Engela and Ohangwena) fall under Engela Health District where the current study took place. Nevertheless, 62.3% of the population in the Ohangwena region resides in the Engela district.^15^ The majority of population (89.9%) lives in rural areas as compared to the 10.1% living in urban areas. The health services in the district are provided by one regional hospital, two health centres and 18 clinics and outreach services posts.

The predominant activities in the region include cattle husbandry and small-scale agriculture. However, this region is prone to natural disasters such as drought and floods that consequently predispose the local residents to famine and waterborne diseases.^15,16^

### Study population and sampling techniques

Engela district in Ohangwena region had a total number of 7828 households. The target population in this study comprised 1004 children under 5 years of age chosen from amongst 530 households. A representative sample of 530 children aged under 5, whose mothers or caregivers consented to participate in the study and who were residing in the area for 1 year, were selected. A multistage cluster sampling method was used and the constituencies were considered as clusters. Sampling was conducted as follows: in the first stage, five constituencies out of seven were randomly selected. In the second stage, the villages were selected using the stratified proportionate sampling method. The researchers purposively included only the households with children aged under 5. Households with at least one child under 5 years were eligible for the study. In households having more than one child under 5, the simple random sampling was conducted to select the child that took part in the study. The researcher, student nurses and health extension workers (HEWs) identified the households with children aged under 5 with the assistance of the village headman and then snowball method (chain referral) was used by asking for further references from houses that have been visited.^17^

### Sample size

The sample size in this study was determined by using the Cochran formula^18^ when the population size is infinite:

$$n=Z^{2*}\frac{p*q}{e^{2}},$$ [Eqn 1]

where *n* = sample size, *z* = desired confidence level 95%, *p* = expected prevalence, *q* = 1–p (expected non-prevalence) and *e* = relative desired precision.

$$\text{Thus},\;n=\frac{1.96^{2*}0.5*0.5}{0.05^{2}}=384.16HH=384\text{HH}.$$ [Eqn 2]

Thirty-eight (38) households (HH) representing 10 % of non-response was added to the sample. Therefore, a minimum of 422 HH was required; nonetheless, we managed to survey 530 households, with a total of 1004 children under 5. From each household, only one child under 5 participated; therefore, a total of 530 children aged under 5 took part in the study.

### Data collection method

A structured questionnaire was developed based on some aspects adopted from the Namibian Demographic Health Survey (NDHS) 2013 and was used for data collection. The questions were modified and amended for cultural specificity to suit the current study.^14^ The questionnaire was then translated from English to the local language by a language expert and then back-translated to English after data collection. The HEWs and student nurses were trained prior to data collection to assist in the process. The researcher, student nurses and HEWs read out the questions to the respondents and filled in the respondents’ answer verbatim as they were given by the respondents and left with the questionnaire when the interview was over.^19^ Data collection was supervised by the researcher who checked for completeness of the collected questionnaire daily.

### Measurements of the variables

The primary outcome variable was the occurrence of diarrhoea within 2 weeks prior to the data collection.

The dependent variable for this study was defined as passage of three or more loose or liquid stools in 24 h.^20,21^ The prevalence of diarrhoea was calculated by dividing the number of children (126) who had an episode of diarrhoea within 2 weeks prior to data collection by the overall number of children (530).

The independent variables included demographic characteristics (children aged under 5, child sex, and mother or caregiver’s age), socio-economic characteristics (residence, educational status, educational level, employment status, household income, number of people per household, number of sleeping rooms, ownership of refrigerator, materials of floor, materials of walls, materials of roof, etc.), health characteristics (immunisation status and nutritional status), access to information (ownership of televisions [TVs], ownership of radios) and environmental characteristics (such as source of drinking water, distance from water source, the type of toilets and sharing of toilets).

In this study, water from pipes and from protected springs and/or wells was considered as an improved source as indicated by Mengistie et al.^7^

### Data quality control

For the purpose of testing validity and reliability of the instrument, a pilot study was conducted on 5% of the sample prior to data collection in the constituency that was not part of the study. The assessment of the reliability and validity of data collection tool found a Cronbach’s alpha value of 0.810; this shows that the data collection items measured the same concept.

Hence, the instrument was modified accordingly based on the outcomes of the pilot study. Prior to data collection, participants (student nurses and HEWs) were trained in the process and continuous supervision was carried out by the main researcher.

### Data analysis

Descriptive statistics were used to summarise the data. Data were presented as frequency distribution tables, consisting of frequencies, percentages and 95% confidence intervals. The chi-square (χ^2^) test was used to test for statistical relationship between independent and dependent variables (prevalence of diarrhoeal disease). The researcher applied logistic regression analysis to identify significant factors associated with the prevalence of diarrheal disease in children aged under 5, and to determine the likelihood of factors that have an impact on the prevalence of diarrhoea. The model containing all the predictors was statistically significant (χ^2^ [16, *N* = 530] = 58.44, *p* ˂ 0.001); this is an indication that the model was able to distinguish between respondents who reported children suffering from diarrhoea and those who did not report the incidence of diarrhoea. We used the International Business Machines (IBM) Statistical Package for Social Science (SPSS) software version 25 to perform this analysis. Variables with statistical significance (*p* < 0.05) in the bivariate analysis were included in the logistic regression to determine factors associated with the prevalence of diarrhoeal disease.

### Ethical consideration

Ethical clearance was obtained from the University of Namibia Research Ethics Committee (ref. no: SON/553/2019). Permission to conduct the study was obtained from the Ministry of Health and Social Services (ref: 17/3/3BF) and the Regional Director for the Ohangwena Region. The principles of the Declaration of Helsinki werw followed and adhered to in conducting the study. Mothers or caregivers were informed about the survey procedures and consent was sought from them prior to interviews.

## Results

### Socio-demographic characteristics of the respondents

A total of 530 children aged under 5 from 530 households were included in the study, with a 100% response rate. Socio-demographic characteristics of the respondent are presented in Table 1. Approximately, 79.8% of respondents were from rural areas whilst only 1% were from the urban area. A total of 29.4% of mothers or caregivers were aged between 18 and 29 years. The median age of the respondents was 38 years. The majority (74.9%) of the mothers or caregivers were unemployed. Almost half (46.4%) of the mothers or caregivers had only primary education. The results showed that majority (93.2%) of the household income ranged between N$ 190 and N$ 2000 per month. Equally, the biggest proportion of the children who suffered from diarrhoea (23.9%) were from households with the lowest income of N$ 190 – N$ 2000. Nonetheless, the observed association between the levels of income and occurrence of diarrhoea was not significant. The mean household size was 8.9 (standard deviation [s.d.]: 5.3) individuals. In addition, results showed that 52.1% of houses were overcrowded and had 5–10 occupants, 25.8% had 10–20 occupants and 3.0% had more than 20 occupants. There was a statistically significant association between the prevalence of diarrhoea and the number of occupants in the households (*p* < 0.05).

**TABLE 1:** Distribution of socio-demographic characteristics of the children under 5 years old and mothers or caregivers related to prevalence of acute diarrhoea.

Variable	Suffered from diarrhoea in past 2 weeks				Total		95% CI of the prevalence of diarrhoea	p*
	Yes		No					
	*n*	%	*n*	%	*n*	%
**Residential area**								
Urban	0	0	5	100.0	5	1.0	00.00; 00.00	0.001*
Informal settlement	49	48.0	53	52.0	102	19.2	43.75; 52.25	
Rural areas	77	18.2	346	81.8	423	79.8	14.92; 21.48	
**Mother or caregiver age group**								
18–30 years	39	25.0	111	75.0	156	29.4	21.31; 28.69	0.001*
31–40 years	47	33.6	93	66.4	140	23.0	29.58; 37.62	
41–50 years	29	23.2	96	76.9	125	23.8	19.61; 26.79	
51–60 years	7	11.1	56	88.9	63	11.9	08.43; 13.77	
≥ 60 years	4	8.7	42	91.3	46	8.7	06.30; 11.10	
**Employment status of mother or caregiver**								
Unemployed	103	26.0	294	74.0	397	74.9	22.27; 29.73	0.107
Employed	10	27.7	34	77.3	44	8.3	23.89; 31.51	
Self-employed	8	19.0	34	81.0	42	7.9	15.66; 22.34	
Farmer	5	10.6	42	89.4	47	8.9	07.98; 13.22	
**Mother or caregiver education level**								
Not educated	24	28.2	61	71.8	85	16.0	24.37; 32.03	0.658
Primary education	58	23.6	188	76.4	246	46.4	19.98; 27.22	
Secondary education	41	21.7	148	78.3	189	27.9	18.19; 25.21	
Higher education	3	30.0	7	70.0	10	19.0	26.10; 33.90	
**Average monthly income per house hold (N$)**								0.281
190–2000	118	23.9	376	76.1	494	93.2	20.27; 27.53	
> 2000– 5000	8	30.8	18	62.2	26	4.9	26.87; 34.73	
> 5000–10 000	0	0	5	100	5	0.9	00.00; 00.00	
> 10 000	0	0	5	100	5	0.9	00.00; 00.00	
**Number of people per household**								0.029*
1-4	35	34.7	66	65.3	101	19.1	30.65; 38.75	
5–10	61	22.1	215	77.9	276	52.1	18.57; 25.63	
10–20	28	20.4	109	79.6	137	25.8	16.97; 23.83	
˃ 20	2	12.5	14	87.5	16	3.0	09.68; 15.32	
**Children’s age under 5**								0.000*
Child’s age (in months)	-	-	-	-	-	-	-	
Mean age (mean ± s.d., years)	23.67 ± 15.11	-	-	-	-	-	-	
0–11	35	33.2	77	68.8	112	21.0	29.19; 37.21	
12–23	44	31.9	94	68.1	138	26.1	27.93; 35.87	
24–35	23	21.9	82	78.1	105	19.8	18.38; 25.42	
36–47	8	8.2	89	91.8	97	18.3	05.86; 10.54	
48–59	16	20.5	62	79.5	78	14.7	17.06; 23.94	
**Sex of children**								0.944
Male	56	23.6	181	76.4	237	44.7	19.98; 27.22	
Female	70	23.9	223	76.1	293	55.3	20.27; 27.53	
**Immunisation status**								0.029*
Up-to-date	101	22.1	355	77.9	456	86.0	18.57; 25.63	
Not up-to-date	25	33.8	49	66.2	74	14.0	29.77; 37.83	
**Weight for age**								0.010*
Normal weight	30	17.0	146	83.0	176	33.2	13.80; 20.20	
Under-weight	96	27.1	258	63.9	354	66.8	23.32; 30.88	

**Total**	**126**	**23.8**	**404**	**76.2**	**530**	**100**	**-**	**-**

s.d., standard deviation.

* , Pearson Chi-Square statistical significant at 0.05

### Hygiene and access to water

As presented in Table 2, more than half (55.3%) of the respondents indicated they had tap water at home followed by 17.0% who indicated that they buy drinking water from neighbours who have tap water at home; additionally, some respondents (12.6%) reported that they get their water from dug wells, 1.5% get it from public taps and the remaining (13.6%) get their water from other sources. About 86% said it takes them < 15 min to reach the source of water. However, these variables were not significantly associated with the occurrence of diarrhoea. The majority of the respondents (75%) indicated that they boil the water before drinking; 64.8% said to have used chlorination. However, 64.9% indicated that sometimes they do nothing. Most households (73.9%) had toilet facilities at home. Amongst households with toilet facilities, 19.2% had improved toilets and 22.9% had shared toilet facilities.

**TABLE 2:** Characteristics of the household assessed and factors associated with childhood diarrhoea.

Variable	Diarrhoea		No diarrhoea		Total		95% CI of prevalence of diarrhoea	p*
	*n*	%	*n*	%	*n*	%		
**Rooms used for sleeping**								0.033*
One	34	30.4	78	69.6	112	21.0	26.48; 34.32	
Two	28	28.3	71	71.7	99	19.0	24.46; 32.14	
Three	20	20.2	79	79.8	99	19.0	16.78; 23.62	
Four and above	36	17.6	168	82.4	204	39.0	14.36; 20.84	
**Commonly used source of drinking water**								0.001*
Tap water	56	19.1	237	80.9	293	55.3	15.75; 22.45	
Public tap	2	25.0	6	75.0	8	1.5	21.31; 28.69	
Buy from private owners	42	46.7	48	53.3	90	17.0	42.45; 50.95	
Dug well	11	16.4	56	83.6	67	12.6	13.25; 19.55	
Others	15	20.8	57	79.2	72	13.6	17.34; 24.26	
**Water treatment**								
*Boil water*								0.036*
Yes	86	21.6	313	78.4	399	75.3	18.10; 25.10	
No	40	30.5	91	69.5	131	24.7	26.58; 34.41	
*Add bleach chorine*								0.012*
Yes	70	55.6	273	67.7	343	64.8	51.37; 59.83	
No	56	30.1	130	69.9	186	35.2	26.19; 34.01	
*Do nothing*								0.216
Yes	76	22.1	268	77.9	344	64.9	18.57; 25.63	
No	50	26.9	136	73.1	186	35.1	23.12; 30.68	
**Time taken to obtain water**								0.294
˂ 15 min	113	24.7	334	75.3	457	86.0	21.03; 28.37	
15–30 min	8	15.1	45	84.9	53	10.0	12.05;18.15	
> 30 min	5	25.0	15	75.0	20	04.0	21.31; 28.69	
**Availability of toilet at home**								
Yes	22	15.5	120	84.5	142	26.8	12.42; 18.58	0.007*
No	104	26.8	284	73.2	388	73.9	23.03; 30.57	
**Type of toilet facility**								0.022*
Improved	17	16.7	85	83.3	102	19.2	13.52; 19.88	
Unimproved	5	12.5	35	87.5	40	7.5	09.68; 15.32	
**Sharing of toilets**								0.825
Yes	4	16.7	20	83.3.0	24	22.9	09.90; 23.49	
No	12	14.8	69	85.2	81 77.1	08.01; 21.59		
**Ownership of television**								0.692
Yes	15	25.9	43	74.1	58	10.9	22.17; 29.63	
No	111	23.5	361	76.5	472	89.1	19.89; 27.11	
**Ownership of radio**								0.005*
Yes	74	20.3	291	79.7	365	68.9	16.88; 23.72	
No	52	31.5	113	68.5	165	31.1	27.54; 35.43	
**Ownership of refrigerator**								0.111
Yes	23	31.1	51	68.9	74	14.0	27.16; 35.04	
No	103	22.6	353	77.4	456	86.0	19.04; 26.16	
**Materials of floor of the house**								0.129
Earth or sand	80	24.4	248	75.6	328	62.0	20.74; 28.06	
Cement or others	42	25.8	121	74.2	163	30.8	22.07; 29.53	
Mud or clay	4	10.5	34	89.5	38	07.2	07.89; 13.11	
**Materials of the exterior walls of the house**								0.001*
Corrugated iron or zinc	69	33.0	140	33.0	209	39.4	29.00; 37.00	
Bricks	25	15.2	139	84.8	164	30.9	12.14; 18.26	
Sticks with mud or dag, reused wood	32	20.8	122	79.2	154	29.0	17.34; 24.26	
Tin	0	0.0	3	07	3	0.6	00.00; 00.00	
**Materials of the roof**								0.002*
Corrugated iron or zinc	81	30.3	186	69.7	267	50.4	26.39; 34.21	
Thatched or palm leaf or grass	42	16.9	207	83.1	249	47.0	13.71; 20.09	
Others	3	21.4	11	78.6	14	02.6	22.27; 29.73	

**Total**	**126**	**23.8**	**404**	**76.2**	**530**	**100**	-	-

CI, confidence interval.

* , Pearson’s chi-square statistically significant: 0.05.

### Prevalence of diarrhoea amongst children under the age of 5 years

Prevalence of diarrhoea amongst children under 5 was estimated by dividing the number of children who reportedly had diarrhoea during the past 2 weeks before the survey by the overall number of children in the sample, which gave an overall prevalence of 23.8%. Children who resided in informal settlement (48.0%; 95% confidence interval [CI] of 43.75; 52.25; *p ≤* 0.001) had a higher risk of developing diarrhoea in the last 2 weeks prior to data collection as compared to those who were from other settlements; the observed difference was statistically significant. The prevalence of diarrhoea was greater amongst mothers or caregivers aged 31–40 years (37.6%; 95% CI of 29.58; 37.62; *p ≤* 0.001); the observed difference was statistically significant. The age for under-5 ranged from 0 to 59 months; the mean age was 23.67 months with an s.d. of 15.11 months. To allow comparison between different age groups, the age of children under 5 was categorised into groups of ˂ 12, 12–23, 24–35, 36–47 and 48–59 months. Analysis stratified by age group showed a high prevalence of diarrhoea amongst children aged between 0 and 12 months (33.2%) and the lowest was between 36 and 47 months (8.2%). The observed difference was statistically significant. More than half of the children were females (55.3%) and around 44.7% were males. The prevalence of diarrhoea related to child’s gender was slightly high (23.9%) among female as compared to male (23.6). However, the difference was not statistically significant (*p* > 0.05). The majority of children (86.0%) received all the required vaccines; nonetheless, 14.0% children’s immunisations were not up-to-date. Children who did not receive all their vaccines (33.8%; 95% CI of 29.77; 37.83; *p ≤* 0.03) had a higher risk of developing diarrhoea as compared to 22.1% who did not develop diarrhoea, and the results were statistically significant.

The majority (66.8%) of the children were reported to be underweight in the past 2 weeks prior to the survey. The findings were statistically significant and children who were underweight had a higher risk (27.1.2%; 95% CI of 23.32; 30.88; *p ≤* 0.01) of suffering from acute diarrhoea than those who had normal weight (23.8%).

### Epidemiology of diarrhoea by types

**FIGURE 1:**
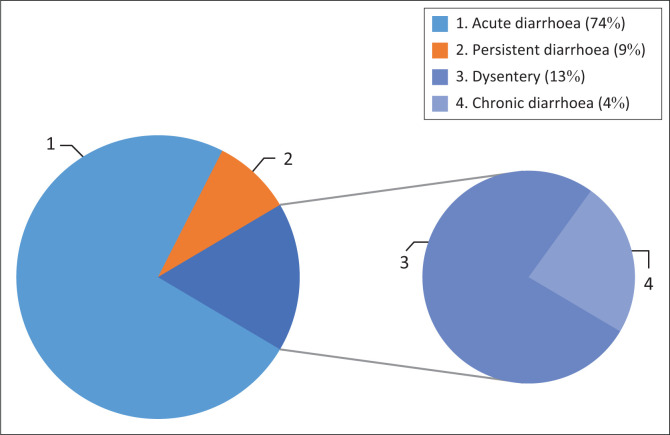
Types of diarrhoea.

Types of diarrhoea were classified as acute diarrhoea (that lasted less than 5–7 days), persistent diarrhoea (that lasted more than 7 days but less than 14 days), chronic diarrhoea (that lasted more than 14 days) and dysentery (when there is blood in stools). Children who were suffering from diarrhoea were classified as suffering from acute diarrhoea (74%), persistent diarrhoea (9%), dysentery (13%) and chronic diarrhoea (4%) (see Figure 1).

**FIGURE 2:**
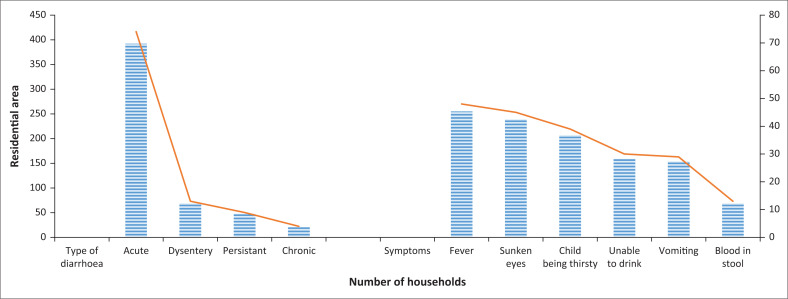
Proportions of types of diarrhoea and accompanying symptoms.

Mothers or caregivers reported children having shown the following symptoms: fever (48%), sunken eyes (45%), child being thirsty (39%), unable to eat or drink (30%), vomiting (24%) and blood in stool (13%) (see Figure 2).

### Epidemiology of childhood diarrhoea by environmental factors

Table 2 highlights environmental characteristic of households related to occurrence of diarrhoea amongst children aged under 5. Related to number of sleeping rooms per household, 39% of respondents indicated that they had four and more sleeping rooms. The number of sleeping rooms was found to be significantly associated with the prevalence of diarrhoea (*p* < 0.05). The majority of respondents (89%) indicated that they had access to improved source of water, and 86% said it takes them < 15 min to reach the source of water. However, these variables were not significantly associated with the occurrence of diarrhoea. Again, the majority of the mothers or caregivers (73.2%) reported that they did not have access to toilet facilities and instead used bushes for such purposes; only a minority (19.2%) had access to improved toilet facilities. Children whose mothers or caregivers indicated not having access to toilets had a significantly increased risk (26.8%; 95% CI: [23.03; 30.57]; (*p* < 0.05) of developing diarrheal disease compared to those who indicated otherwise (16.7% and 12.5%, respectively). A small percentage (15.2%) of participants indicated to have shared toilets with neighbours. A minority of the mothers or caregivers indicated owning a television (10.9%) and refrigerator (14.0%). However, these variables were not significantly associated with diarrhoeal occurrence. On the other hand, more than half of the participants (68.9%) claimed that they owned a radio. Nonetheless, owning a radio was found to be significantly associated with the occurrence of diarrhoea (*p* < 0.05). The majority of the mothers or caregivers (62.0%) indicated that the floor in their house was made up of sand; notwithstanding, this was not significantly associated with the occurrence of diarrhoea. Again, the mothers or caregivers indicated that the walls of their houses were constructed with corrugated iron or zinc (39.4%); on the other hand, 50.4% indicated that roofs of their houses were covered with corrugated iron or zinc as well. This was significantly associated with occurrence of diarrhoea amongst children aged under 5.

**FIGURE 3:**
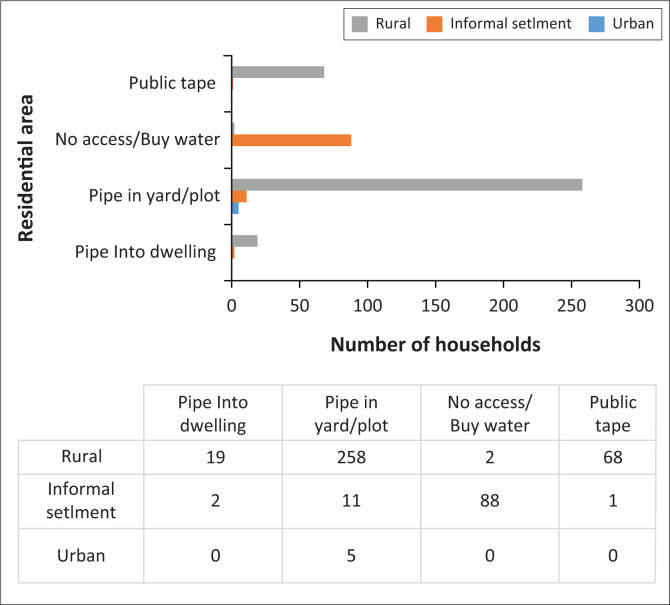
Households’ sources of drinking water.

The source of drinking water was significantly related to diarrhoeal disease (*p* ≤ 0.001). Figure 3 shows that 86.3% of people residing in informal settlements did not have access to piped water and they bought water from those who had piped water in their yards, or made use of dug wells.

### Factors associated with acute diarrhoea amongst children aged under 5 years

Table 3 shows the factors influencing diarrhoeal prevalence. For this purpose, direct logistic regression was performed to assess the impact of a number of factors on the likelihood that the mothers or caregivers would report that their child had diarrhoea in the past 2 weeks prior to the survey. The model contained nine independent variables (residential area, source of drinking water, type of toilet facility, type of floor, age category of children aged under 5, education level of mother or caregiver, employment of mother or caregiver, under-five immunisation and nutritional status). The full model containing all predictors was significant χ^2^ [19 *N* = 524] = 66.192, (*p* ˂ 0.001), indicating that the model was able to distinguish between the respondents who did not and those who reported that children aged under 5 had diarrhoeal episodes in the 2 weeks preceding the survey. The model as a whole explained between 11.9% (Cox and Snell *R*-squared) and 17.9% Nagelkerke *R*-squared) of the variance in the prevalence of acute diarrhoea, and correctly classified 78.4% of the cases. As shown in Table 3, only three independent variables made a unique statistically significant contribution to the model (rural residential area, the age category of children aged under 5 and the nutritional status of these children). The strongest predictor for children aged under 5 suffering from diarrhoea in the 2 weeks was place of residence (informal settlement), recording an OR of 36.42. This indicates that children residing in an informal settlement were over 36.42 times likely to suffer from diarrheal disease than those who were living in rural areas, controlling for all other factors in the model. On the other hand, the OR of 0.31 for children living in rural areas was less than 1, indicating that for every increase in living in rural areas, the children aged under 5 were 0.31 times less likely to suffer from acute diarrhoea. Odds ratio of 2.15 for age categories of 36–47 months indicated that children in this age range were over two times more likely to suffer from diarrhoea as compared to children falling in other age groups. Availability of toilet facilities was found to be significantly associated with prevalence of diarrhoea *p* ˂ 0.007). The OR of 0.50 having a toilet at home was less than 1, indicating that households with toilets were 50 times less likely to report the incidence of diarrhoea in children aged under 5, controlling for other factors in the model.

**TABLE 3:** Logistic regression – Factors associated with diarrhoea.

Variable	Wald	df	Sig	Exp(B)	95% CI for Exp B	
					Lower	Upper
**Residence**	16.64	2	0.00*	-	-	-
Informal settlement	0.00	1	0.01*	36.42	0.00	-
Rural	16.64	1	0.00*	0.31	0.17	0.54
**Materials used for walls**	3.33	3	0.34	-	-	-
Bricks	0.00	1	1.00	0.00	0.00	-
Corrugated iron or zinc	0.00	1	1.00	0.00	0.00	-
Sticks with mud or clay or reused wood	0.00	1	1.00	0.00	0.00	-
**Materials used for roof**	1.62	2	0.45	-	-	-
Corrugated iron or zinc	0.24	1	0.62	0.70	0.17	2.90
Thatched or palm leaf or grass	0.00	1	0.97	1.03	0.25	4.21
Rooms for sleeping	0.03	1	0.86	1.02	0.81	1.29
**Type of toilet facility**	1.71	2	0.43	-	-	-
Improved	0.62	1	0.43	1.30	0.68	2.49
Not improved	1.31	1	0.25	1.82	0.65	5.04
Number of people per household	1.22	1	0.27	0.91	0.76	1.08
**Child’s age category in months**	13.58	4	0.00*	-	-	-
˃ 12	3.06	1	0.08	0.52	0.25	1.08
12–23	4.84	1	0.03*	0.45	0.22	0.92
24–35	0.88	1	0.35	0.69	0.32	1.49
36–47	2.53	1	0.11	2.13	0.84	5.52
**Mothers’ or caregivers’ age category**	2.08	1	0.15	1.16	0.95	1.41
Ownership of radio	0.18	1	0.67	0.89	0.17	2.90
**Immunisation status up to date**	0.12	1	0.73	1.12	0.60	2.06
**Have a toilet at home (1)**	7.15	1	0.007*	0.50	0.30	0.83
**Nutritional status of children aged under 5**	7.72	1	0.01*	2.047	1.24	3.39

CI, confidence interval; sig., *p*-value; *df*, the degree of freedom.

Variable(s) entered on step 1: residential area, employment, educational level of mother or caregiver, age category of children under 5, nutritional status of children under 5, source of drinking water, type of toilet facility, type of floor, immunisation status of children under 5 and having a toilet at home.

Based on a child’s nutritional status, an OR of 2.05 indicates that children who were underweight were over two times more likely to experience diarrhoea than those with normal weight.

## Discussion

The current study provides the prevalence of diarrhoeal diseases (recall period: 2 weeks) and its determining factors amongst children under 5 in Engela district. The study found that the prevalence of diarrhoea in children under the age of 5 years was 23.8%. This prevalence is slightly lower as compared to studies conducted in other countries. In fact, a 26.7% prevalence of diarrhoeawas found in Nyarungenge district in Ruanda, 26% in Mbour Senegal^6,8^ and a prevalence of 31.0% was found in Arba Minch district, Ethiopia.^22^ On the contrary, the prevalence found in this study was higher as compared to a cross-sectional study conducted in Dale district, Sidama Zone, South Ethiopia, which showed a prevalence of 13.6%. Also, this was very high compared to the countrywide prevalence of 17% as reported in the Demographic and Health Survey (DHS).^14^ High prevalence in the current study was observed in the dry season between January and March; similar findings are reported in other countries, with most diarrhoea cases and death cases occurring between February and March because of rotavirus infection.^6^

On the other hand, the disparity in prevalence could be related to seasonal variations. The current study was conducted during a dry season between January 2019 and March 2019; during water scarcity, people use water from polluted sources of water that is in most cases utilised for washing cloths and bathing only. However, there is a need to explore the influence of climatic parameters and diarrhoea seasonality to be able to more effectively prevent and manage diarrhoea. The majority (86%) of the inhabitants in the informal settlement indicated that they do not have access to tap water and that they buy from those who have tap water within their premises. The majority of mothers or caregivers (44.9%) reported taking their children to the hospital when suffering from diarrhoea. However, 25.8% stated that they would first treat their children at home prior to taking them to hospital. Literature indicates that most mothers seek health services when home management is failed.^23^ Practices indicated as part of diarrhoea management in children under 5 were as follows: mother (20%) and father (8.8%) going for perineal cutting, a child taken to a traditional healer (11.2%), mother stops breastfeeding the baby (3.2%) and a child taken for prayers (4%). However, with a higher percentage (93%) of participants having a low income of N$190 – N$2000 per house, buying water might not be practical. Furthermore, the prevalence of diarrhoea was significantly associated with what was commonly used as a source of drinking water for members of the household.

Results of this study indicated the place of residence to be statistically associated with prevalence of diarrhoea. Children residing in informal settlements were over 36.42 times likely to suffer from diarrheal infections. On the other hand, an OR of 0.31 for children living in rural areas was less than 1, indicating that for every increase in living in rural areas, the children under 5 were 0.31 times less likely to suffer from acute diarrhoea. These findings could be related to the fact that majority of the respondents from rural areas indicated having tap water in their plots (74.4%) and 3.5% indicated having indoor taps. On the other hand, out of the respondents residing in informal settlement, only 10.8% indicated they had water in their plots, with a minority (2%) mentioning about indoor taps. However, even if the household has a water connection at home, because of recurrent interruption in water supply inhabitants might need to go to dug wells or seek other sources of water supply.

Moreover, it is reported that water supply or treatment statistically played a significant role in reducing the risk of diarrhoea in children aged under 5.^6,10^ This study also found that the child’s age group was significantly associated with the prevalence of diarrhoea. Diarrhoea was found to be more prevalent (34.9%) amongst children in the age group of 12–23 months. These findings were in contrast to results from Mbour Senegal, where the prevalence was found to be high in the age group of 24–59 months.^6^ However, the findings were in line with findings from a study conducted by Sarker et al.^4^

The findings of this study regarding the association between the nutritional status of children under 5 years and the occurrence of diarrhoea are similar to the results of the study conducted by Barbatha and AlEzzi in Al-Mukalla, Yemen.^24^ Hence, there is a need to reinforce primary healthcare nutritional programmes and conduct community-based education on nutrition for the mothers and caregivers, keeping in mind the locally available foods.

Hence, there is a need to reinforce primary healthcare nutritional programmes and conduct community-based education on nutrition for the mothers and caregivers, keeping in mind the locally available foods. These findings imply an increase in health financing - particularly, the implementation of nutritional programmes at primary healthcare facilities.

Furthermore, the socio-economic and accesses to information characteristics were significantly associated with the prevalence of diarrhoea; these findings are in line with a study by Sarker et al.^4^ They found that households that had access to radio and television were most likely to be informed and seek care from public facilities for childhood diarrhoea. Using mass media has a positive impact on behavioural change in terms of health-seeking practices. Therefore, the community health nurses need to strengthen health education on hygiene and nutrition using mass media, such as radio and television, and the HEWs need to create awareness by going from household to household and through churches and locally held meetings to cater to the mothers or caregivers who might not have access to radio and television.

The availability of toilet facilities was found to be significantly associated with the prevalence diarrhoea. The OR of 0.50 of having a toilet at home was less than 1, indicating that households with toilets were 50 times less likely to report an incidence of diarrhoea in children under 5, controlling for other factors in the model. According to a study conducted by Bitew et al. on childhood diarrhoeal morbidity and sanitation predictors in a nomadic community, unavailability of any type of toilets (Adjusted Odd Ratio (AOR) = 2.28, 95% CI = 1.05, 4.97) and presence of human excreta in the compound (AOR = 11.39, 95% CI = 2.10, 61.79) were amongst the factors found to be statistically associated with the prevalence of childhood diarrhoeal disease.^25^ Consequently, the regional council and the district commissioners need to strengthen and accelerate the provision of water and sanitation facilities.

Future research studies will be needed to assess food security in the region and understand how the preparation of locally available food for children under 5 is done by the mothers or caregivers. Also, evaluation of the knowledge, attitude and practices of mothers or caregivers regarding the prevention and management of acute diarrhoea is required.

External and internal validity may be affected in some respects because of limitations presented in the study. There was a very small number of participants that were from urban areas as compared to rural and informal settlements; equal numbers of participants could be collected to make a better comparison of findings. Moreover, mothers or caregivers were asked to recall previous events. Consequently, the potential effects of recall bias on our results cannot be overlooked.

## Conclusion and recommendation

The prevalence of diarrhoea in children aged under 5 remains a public health problem in the district. The prevalence of diarrhoea was found to be higher as compared to the national level. Children whose immunisation was not up-to-date, those who were residents from informal settlements, living in houses with walls made up corrugate iron, and those who did not have access to piped water and toilets were significantly affected by diarrhoea. In conclusion, our study revealed results that show a high prevalence of diarrhoea (23.8%) as compared to the national figures (17%). However, logistic regression revealed factors such as residential area, age category and nutritional status of children aged under 5 as independent predictors of diarrheal disease.

Therefore, we recommend provision of safe water and toilets, and improved literacy. Health education on environmental sanitation should be strengthened in order to be able to decrease childhood diarrhoea. Significant attention should be given to informal settlements where diarrhoea was common. Health service providers should provide regular health education sessions to increase the knowledge of mothers about diarrhoea and its misconception.

For public health actions and interventions regarding the epidemiology of diarrhoea, the Ministry of Health and Social Services and other stakeholders should make provision of water purification sachets as well as increase the awareness on sanitation. Furthermore, the government should ensure provision of safe water at informal settlements and also ensure provision of toilets. The findings contain useful information that can be used by the existing national programme for the fight against diarrhoea.
