# Erratum: Epidemiology and factors associated with diarrhoea among children under five years of age in the Engela District in the Ohangwena Region, Namibia

**DOI:** 10.4102/phcfm.v16i1.4349

**Published:** 2024-01-24

**Authors:** Maria F. Bauleth, Honore K. Mitonga, Lusia N. Pinehas

**Affiliations:** 1 School of Nursing, Faculty of Health Sciences, University of Namibia, Oshakati, Namibia; 2 School of Public Health, Faculty of Health Sciences, University of Namibia, Oshakati, Namibia

In the published article, Bauleth MF, Mitonga HK, Pinehas LN. Epidemiology and factors associated with diarrhoea amongst children under 5 years of age in Engela district in the Ohangwena region, Namibia. Afr J Prim Health Care Fam Med. 2020;12(1), a2361. https://doi.org/10.4102/phcfm.v12i1.2361, there was an error with the conclusion statement and keywords, as the keywords belonged to another article.

## The original incorrect

**Conclusion:** epidemiology; factors; diarrhoea; under-5 years children; Engela district; Ohangwena region; Namibia.

**Keywords:** COVID-19; pandemic; Nigeria; family physicians; frontline.

## The revised and updated

**Conclusion:** This study identified the need to develop and intensify strategies to improve the health of under five-year-old children in the region, such as the provision of safe water, toilet facilities and environmental sanitation.

**Keywords:** Epidemiology; factors; diarrhoea; under five year’s children; Engela District; Ohangwena Region; Namibia.

The publisher apologises(s) for this error. The correction does not change the study’s findings of significance or overall interpretation of the study’s results or the scientific conclusions of the article in any way.

